# Supplementary material for: Impact of inter- and intra-individual variation, sample storage and sampling fraction on human stool microbial community profiles
Source: PeerJ. 2019 Jan 11;7:e6172. doi: 10.7717/peerj.6172 (PMC6330951; doi:10.7717/peerj.6172)
Supplement: Supplemental Information 2 — Respective figure legends are underneath each figure. [file peerj-07-6172-s002.pdf]

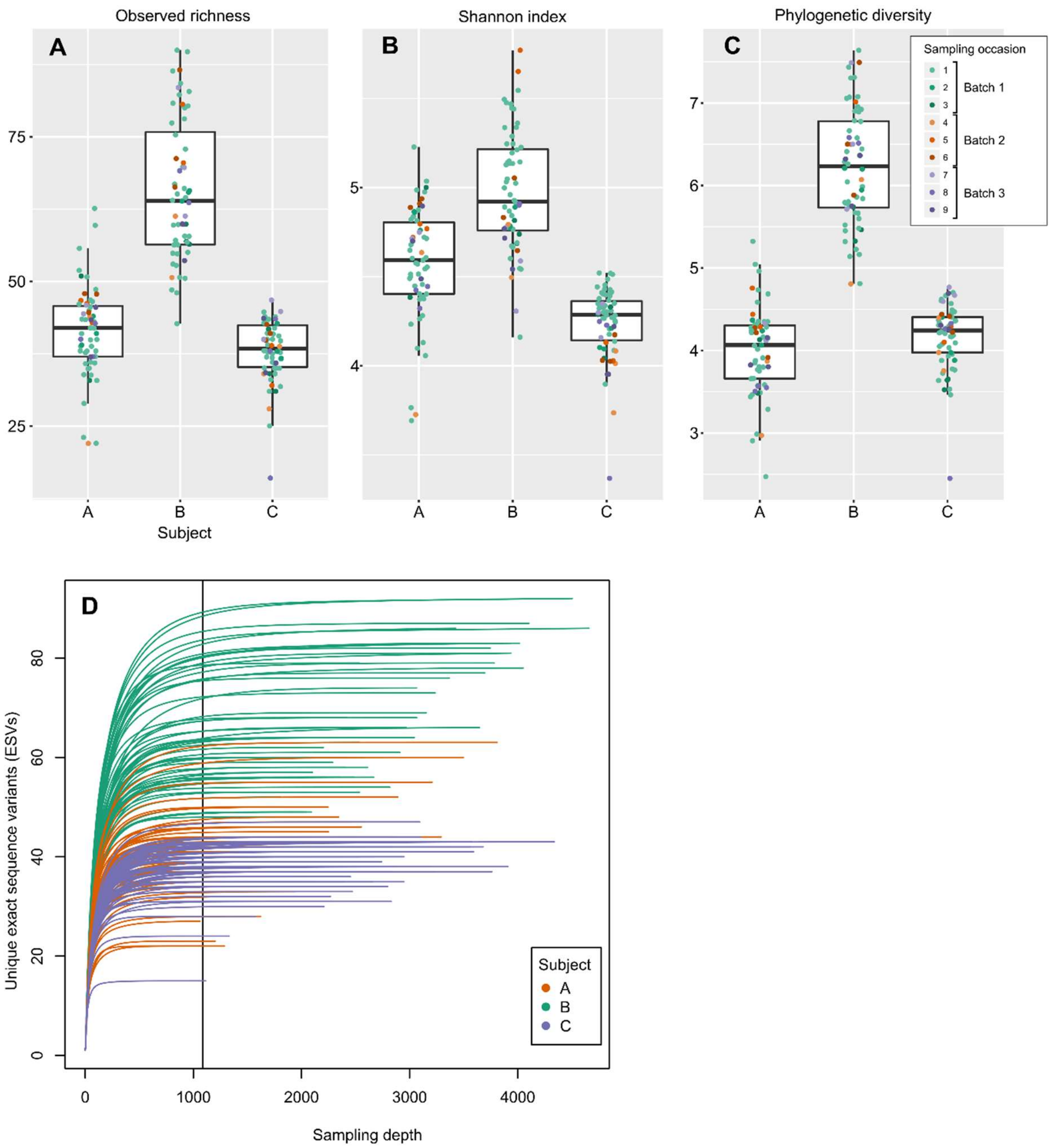

Figure S1. Alpha diversity metrics of stool communities sampled from three donors over 72 days. **A**. Observed richness; **B**. Shannon diversity index; **C**. Faith's phylogenetic diversity; **D**. rarefaction curve showing number of unique exact sequence variants (ESVs) detected vs sampling depth. In panels A-C, each point represents a sample, colour coded by sampling occasion (refer to Fig. 1 for time line). The bold centre line within the boxplot rectangles represents median values, and the two ends of the rectangle represent upper and lower quartiles. The upper whisker extends to the highest value within 1.5x the interquartile range above the upper quartile, whereas the lower whisker extends to the lowest value within 1.5x the interquartile range below the lower quartile. All diversity metrics were calculated using QIIME2 v2017.12. Values presented here are based on a normalised sampling depth of 1086 reads per sample as indicated by the black vertical line in panel D.

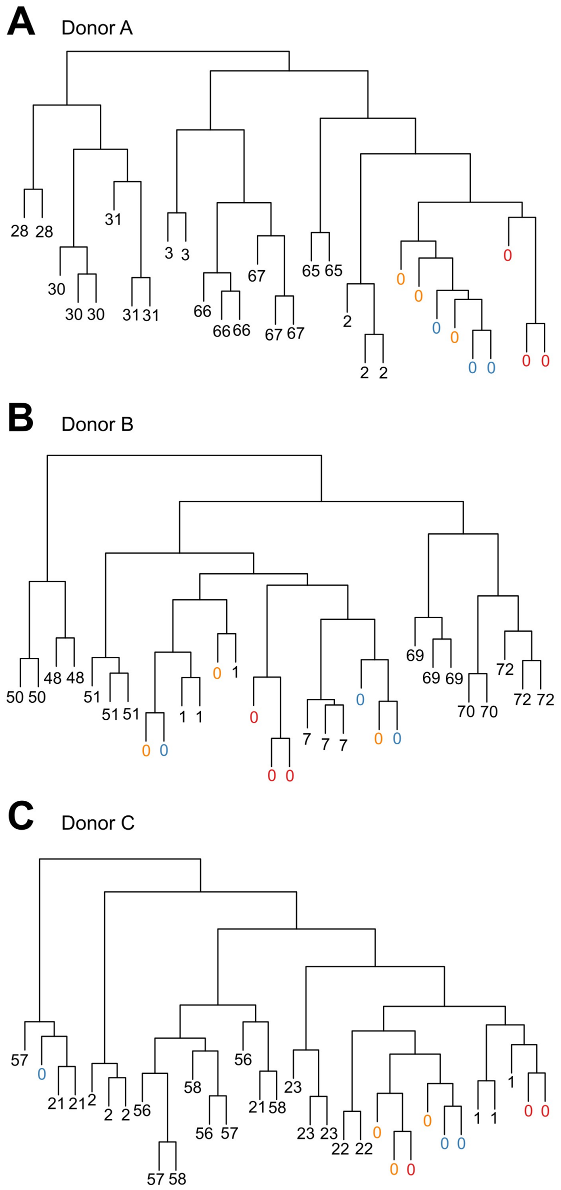

Figure S2. Complete linkage hierarchical clustering of stool community composition from three donors collected over 72 days. Clustering was performed on centered log-ratio transformed community composition counts data. **A**. Donor A; **B**. Donor B; **C**. Donor C. Numbers represent days stools were collected after the first stool collection occurred on day 0; identical numbers represent biological replicates. Samples from day 0 are colour coded red, orange or blue to indicate triplicates samples taken from three different fractions of the same stool to assess effects of subsampling stools on community composition (detailed in Results section: *Effect of sampling different stool fractions on community composition*).

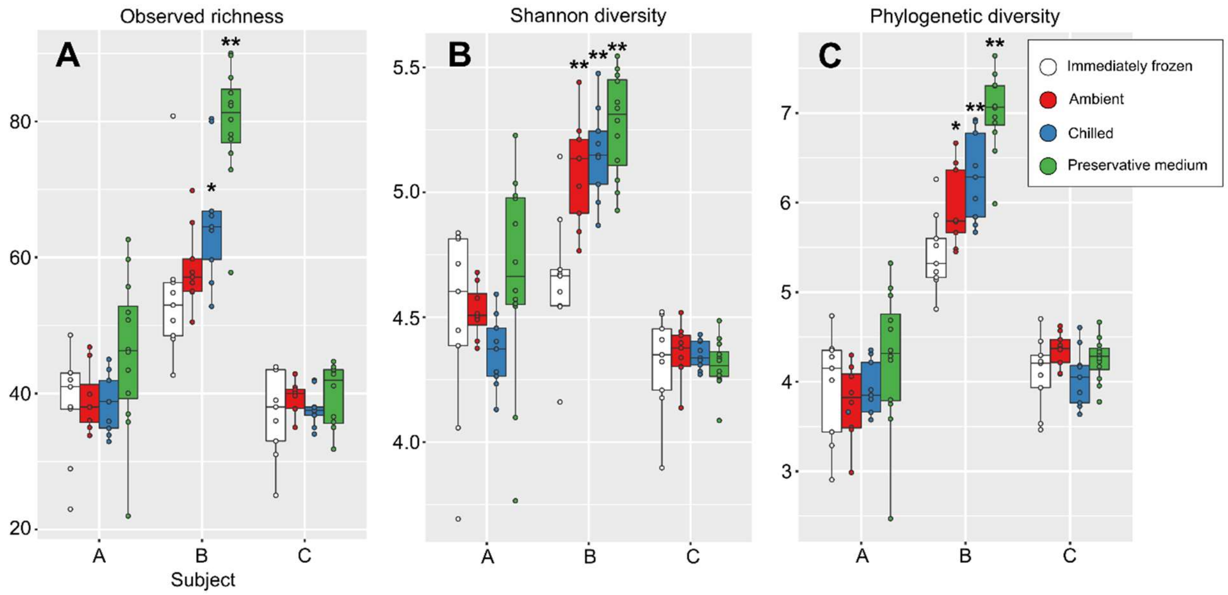

Figure S3. Alpha diversity metrics of stool communities sampled from three donors from one time point (day 0; see Fig. 1 for time line). **A**. Observed richness; **B**. Shannon diversity index; **C**. Faith's phylogenetic diversity. Each point represents a sample and is colour coded by storage condition: white, immediately frozen at -80 ℃; red, ambient temperature; blue, chilled; green, stored in preservative medium at ambient temperature. The centre line within the boxplot rectangles represents median values, and the two ends of the rectangle represent upper and lower quartiles. The upper whisker extends to the highest value within 1.5x the interquartile range above the upper quartile, whereas the lower whisker extends to the lowest value within 1.5x the interquartile range below the lower quartile. All diversity metrics were calculated using QIIME 2 v2017.12. Kruskal-Wallis tests were performed to assess whether the three storage conditions resulted in changes in each of the three alpha diversity metrics (*: p <0.05, **: p <0.01).

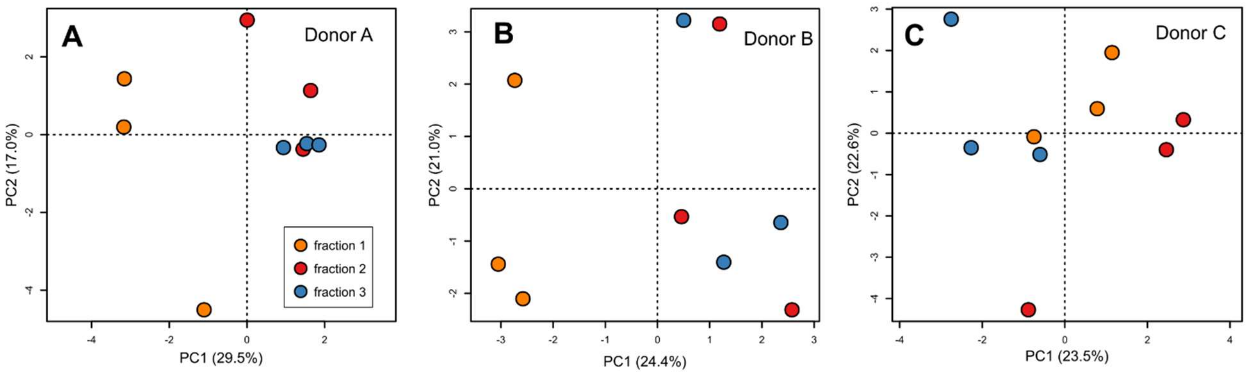

Figure S4. Principal component ordination of microbial community composition in stools collected from three donors. **A**. Donor A. **B**. Donor B. **C**. Donor C. One stool from each donor was subsampled three times to assess the effect of subsampling on microbial community profiles. Each point represents one biological replicate from one of three subsampled fractions and are colour coded: orange, fraction 1; red, fraction 2; blue fraction 3.

Table S1. Sample metadata.

Table S2: ESVs enriched in preservative medium samples determined using GLM (three subjects combined).

Table S3: ESVs enriched in preservative medium samples determined using GLM (subjects analysed separately).

Table S4. Effect of storage temperature and duration on stool community composition.

Table S5. ESVs enriched/depleted over 24 hours in ambient and chilled stool samples.

Table S6. Effect of subsampling stools on community composition.
